# Peer Review of “Waiting Time and Patient Satisfaction in a Subspecialty Eye Hospital Using a Mobile Data Collection Kit: Pre-Post Quality Improvement Intervention”

**DOI:** 10.2196/40574

**Published:** 2022-08-09

**Keywords:** Waiting time, waiting list, patient satisfaction, quality improvement, clinical audit, ophthalmology, patient-centered care

*This is a peer-review report submitted for the paper “Waiting Time and Patient Satisfaction in a Subspecialty Eye Hospital Using a Mobile Data Collection Kit: Pre-Post Quality Improvement Intervention.”*

## Round 1 Review

I have completed the statistical review of this manuscript [1], which is well-organized and presented. However, the following suggestions will help improve the quality of this manuscript.

1. Is it a proof-of-concept–type study? Kindly add the time period of this study.
2. Kindly do not use word “subjects” for study participants. You can simply use either “participants” or “patients.”
3. No power calculation rationale was provided in this report, so these results cannot be generalized.
4. Authors must include statements regarding the statistical software to perform data analysis and what level of statistical significance was used for hypothesis testing.
5. Authors must add more clarity to the “Logistic regression with reported…” statement as odds ratios with 95% confidence intervals are calculated from the logistic regression. What is the point of margins plot in this case? What other covariate were adjusted in the logistic
6. Table 1, the cohabiting group can be merged with the married group. Add “years” in brackets next to “Age.” Arrival time can also be sensibly presented with fewer meaningful categories.
